# Unlocking the Karyological and Cytogenetic Diversity of *Iris* from Lebanon: *Oncocyclus* Section Shows a Distinctive Profile and Relative Stasis during Its Continental Radiation

**DOI:** 10.1371/journal.pone.0160816

**Published:** 2016-08-15

**Authors:** Nour Abdel Samad, Magda Bou Dagher-Kharrat, Oriane Hidalgo, Rana El Zein, Bouchra Douaihy, Sonja Siljak-Yakovlev

**Affiliations:** 1 Faculté des Sciences, Département Sciences de la Vie et de la Terre, Laboratoire Caractérisation Génomique des Plantes, Campus Sciences et Technologies, Université Saint-Joseph, Mar Roukos Mkalles, Lebanon; 2 Ecologie, Systématique, Evolution, UMR 8079 Univ. Paris-Sud, AgroParisTech, Université Paris-Saclay, Bat. 360, 91405 Orsay, France; 3 Royal Botanic Gardens, Kew, Richmond, Surrey, TW9 3AB, United Kingdom; Università di Pisa, ITALY

## Abstract

Despite being an important target of conservation concern and horticultural interest, Lebanese irises yet have a confusing taxonomic history and species’ delimitation is often considered problematic, more especially among royal irises (*Iris* section *Oncocyclus*). Indeed, these irises of exceptionally large and spectacular flowers have radiated across Caucasus and eastern Mediterranean giving rise to a number of strict endemic taxa, many of them being considered under threat. Whilst efforts have mostly focused on clarifying the evolutionary relationships in the group based on morphological and molecular data, karyological and cytogenetic characters have been comparatively overlooked. In this study, we established for the first time the physical mapping of 35S rDNA loci and heterochromatin, and obtained karyo-morphological data for ten Lebanese *Iris* species belonging to four sections (*Iris*, *Limniris*, *Oncocyclus* and *Scorpiris*). Our results evidenced distinctive genomic profiles for each one of the sections, where *Oncocyclus* irises, while having the lowest chromosome numbers, exhibit both the highest number of 35S loci and CMA3+ sites. The continental radiation of royal irises has been accompanied by a relative karyological and cytogenetic stasis, even though some changes were observed regarding karyotype formula and asymmetry indexes. In addition to that, our results enabled taxonomic differentiation between *I*. *germanica* and *I*. *mesopotamica*–two taxa currently considered as synonyms–and highlighted the need for further studies on populations of *I*. *persica* and *I*. *wallasiae* in the Eastern Mediterranean Region.

## Introduction

The genus *Iris* L. includes about 280 species distributed across the temperate region of the Northern Hemisphere [1]. Recurrent hybridization, which has given rise to a myriad of garden forms [2] has also largely contributed to make this genus the largest and most complex of Iridaceae [3]. The classification of *Iris* is indeed a difficult task to tackle; botanists and taxonomists are still far from reaching a consensus on this issue. This problem is certainly reflected through the different subgeneric and sectional classifications established on the basis of morpho-anatomical features, ecological and cytogenetic traits [4,5,6,7]. The most recent taxonomic revision [5] recognizes six subgenera: *Nepalensis* Dykes, *Xiphium* (Miller) Spach, *Scorpiris* Spach, *Hermodactyloides* Spach, *Iris* L. and *Limniris* Tausch. According to Mouterde [8], only the four latter subgenera occur in Lebanon. The subgenus *Iris* is represented by two sections: *Iris* L. with two taxa and *Oncocyclus* (Siemssen) Baker with seven taxa, while the subgenus *Limniris* is represented by two taxa, the subgenus *Hermodactyloides* with one taxon and subgenus *Scorpiris* with two taxa.

This study focused on *Iris* section *Oncocyclus*–the royal irises–which includes numerous strict endemic species. These irises are native to the Near East, especially the Caucasus region, eastern Turkey, Syria, Lebanon and Israel and extend to the Negev Desert. In the east they are found in Iraq, Iran and Afghanistan [9]. *Oncocyclus* section includes 33 species (up to 45 taxa considering subspecies, forms and varieties), which are all regional endemics [10]. In the eastern Mediterranean region, they grow in disjunctive populations separated by short geographical distances [11]. In a recent study, Wilson *et al*. (2016) [12] suggested the Caucasus as the ancestral area for *Oncocyclus* section and the Eastern Mediterranean region as an important area of diversification. From a nomenclature and systematic point of view, *Oncocyclus* section is particularly challenging within Iridaceae [5] and species concept has already been debated [13]. Taxonomic circumscription has been based on minor differences in plant size, leaf shape and flower color. It appears that many local forms have been considered as species, which increased the number of taxa. In addition, the sympatric distribution of some species and interfertility among *Oncocyclus* species [14] could have led to a large number of hybrids, often recognized as full species.

Over the years, taxonomic treatment of the section *Oncocyclus* has experienced a ‘lumping’ change. From 65 species considered by Avishai in 1977 [15], Mathiew in 1989 [5] proposed 41 species and in 1997 Rix [10] proposed only 30. Some sympatric species were recognized as hybrids, but the grouping trend affected mainly the Caucasian species, and no complete or inclusive taxonomic revision of *Oncocyclus* section in the Levant has been carried out to date.

**Fig 1:**
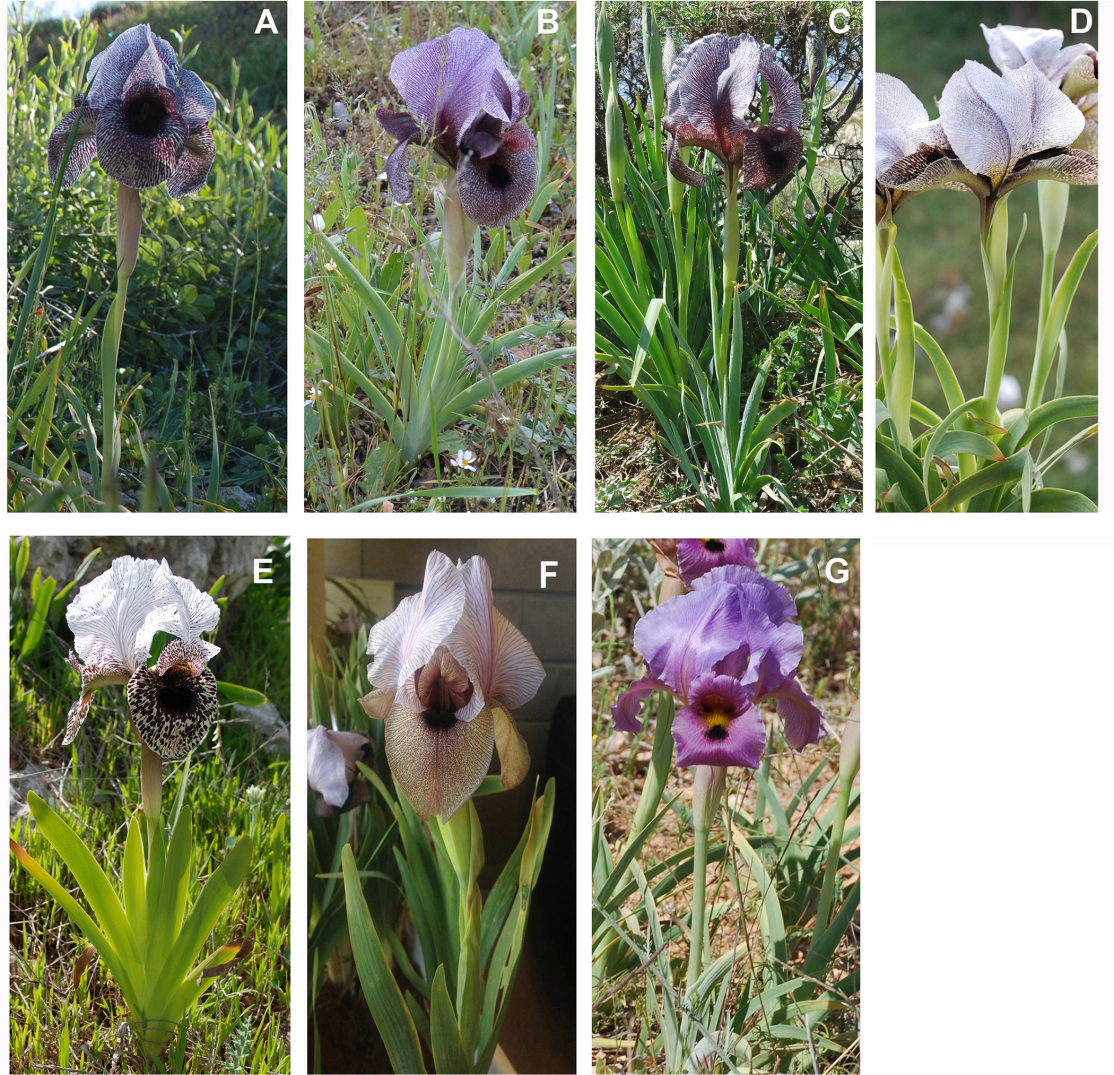
Diversity of Lebanese royal irises, illustrated by some of the studied taxa. A: *Iris sofarana* subsp. *sofarana*, B: *I*. *sofarana* subsp. *kasruwana*, C: *I*. *cedreti*, D: *I*. *westii*, E: *I*. *bismarckiana*, F: *I*. *lortetii*, G: *I*. *antilibanotica*. Photographs from M. Bou Dagher-Kharrat

The Lebanese *Oncocyclus* irises are represented by seven taxa (Fig 1) of which four are considered to be strict endemics [11]: *I*. *cedreti* Dinsmore ex Chaudhary, *I*. *sofarana* Foster subsp. *kasruwana* (Dins.) Chaudhray, *I*. *sofarana* subsp. *sofarana* and *I*. *westii* Dinsmore. They occur in distinct populations across the Mount Lebanon chain [10] between 1200 and 2000 m of altitude. The three other Lebanese *Oncocylus* species are *I*. *bismarckiana* Regel (Lebanon, Syria and Israel) and *I*. *lortetii* Barbey ex Boiss. (Lebanon and Israel) and *I*. *antilibanotica* Dinsm. (Lebanon and Syria).

The botanical descriptions of the endemic Lebanese *Oncocyclus* species, that have been written more than 30 years ago on the basis of a limited sample size, provide however a good illustration of the variability found in populations nowadays [16]. *Oncocyclus* irises are morphologically very close and may be confused because they all have different variants with similar colors, more or less dark purple [5,8,9,17,18]. It is worth noting that the differentiation between *I*. *sofarana* subsp. *sofarana*, *I*. *sofarana* subsp. *kasruwana* and *I*. *westii* according to Mouterde, is only based on the size of the individuals and the shade of standards color.

From a conservation point of view, *Iris* genus includes 53 taxa that are threatened worldwide, of which 29 belong to the *Oncocyclus* section [19]. *Oncocyclus* irises are narrow endemic and of high conservation priority in Lebanon [20,21]. These plants with conspicuous flowers are threatened by intensive collection and habitat destruction. In Lebanon, conservation efforts are now undertaken in order to conserve *Iris* populations through legislation and establishment of micro-reserves [22]. Based on their complete self-incompatibility, fertilization of *Oncocyclus* irises relies on pollination by night-sheltering solitary male bees [23,24,25,26] and Honey bees as frequent diurnal visitors [27]. Nevertheless, pollination is dramatically hindered by the intensive use of pesticides on the adjacent agriculture lands (Bou Dagher-Kharrat, personal observation). Even though they show a dense clonal growth, populations can be sometimes restricted to few dozens of individuals.

Rescue from possible extinction could be performed through the reinforcement of populations by introducing individuals from adjacent populations. In this regard, defining species and differentiating taxa is a crucial and a prerequisite in order to define conservation units. Indeed, conservation strategies require knowing which units (species, subspecies, or populations) need to be preserved and how unique they are.

Wilson *et al*. [12] used in 2016 plastid and nuclear DNA sequences to infer *Oncocyclus* phylogenetic relationships. They found that the species diversified in isolation especially in the Eastern Mediterranean region where populations are scattered on the mountains across rocky hillsides and steppes. However, continental radiation of the royal irises did not involve necessarily chromosome rearrangements, since chromosome number (2n = 20) and their karyotype feature remained basically unchanged throughout *Oncocyclus* taxa [15]. Could molecular cytogenetic bring more insight into this complex *Iris* group?

The physical mapping of rDNA and the distribution of heterochromatin turn out to be a source of chromosomal markers for identifying several chromosome regions [28,29,30,31,32,33] and provides according to Dobigny et al. “valuable information on homologies between chromosomal segments, mainly between closely related species” [34,35,36]. In addition, this set of markers may constitute a phylogenetic tool to detect genome evolution during speciation [37,38,39]. Molecular cytogenetics allowed indeed to detect interesting variation in the rDNA markers as revealed by fluorescent *in situ* hybridization (FISH) patterns in *Iris* subgenus *Xiphium* and evidenced a species-dependent pattern of rDNA sites [40]. However, such studies on the *Iris* genus remain scarce.

The present study combines classical karyological approach and physical mapping of heterochromatin and rRNA genes in order to characterize the genome organization of ten Lebanese *Iris* taxa. The main objective of this study was to contribute to the understanding of phylogenetic relationships, more particularly for the species of *Oncocyclus* section, by checking possible cytogenetic differences between the investigated taxa. For this purpose, besides classical karyological approach, karyotypes were characterized using molecular cytogenetic techniques: FISH for physical mapping of 18S-5.8S-26S (35S) and 5S rRNA genes and fluorochrome banding for distribution patterns of GC-rich heterochromatin regions.

## Material and Methods

### Plant material

In order to obtain fresh root meristems for chromosome preparations, rhizomes of ten *Iris* taxa were collected from ten Lebanese natural populations. Their geographical origins are presented in Table 1.

**Table 1:** Geographical origin (locality and altitude) and flowering date of the investigated taxa.

Subgenus	Section	Taxa	Locality*	Flowering date**	Altitude
*Iris* L.	*Oncocyclus (Siemssen)* Baker	*I*. *antilibanotica* Dinsm.	Khreibeh-Baalback	April-May	1337 m
		*I*. *bismarckiana* Damman & Sprenger	Sarada	March-April	435 m
		*I*. *cedreti* Dinsm.	Bcharreh	May-June	1900 m
		*I*. *lortetii* Barbey ex Boiss.	Mays El Jabal	April-May	640 m
		*I*. *sofarana* subsp. *sofarana* Fost	Falougha	April-May	1640 m
			Hazzerta	April-May	1530 m
		*I*. *sofarana* subsp. *kasruwana* (Dinsm.) Chaudh.	Ehmej	April-May-June	1217 m
		*I*. *westii* Dinsm.	Tawmet Jezzine	April-May	1300 m
	*Iris* L.	*I*. *mesopotamica Dykes*	Mrouje	April-May	1200 m
*Limniris* Tausch	*Limniris* Tausch	*I*. *unguicularis Poiret* var. *cretensis* Dinsm.	Baadaran	April-May	1100 m
*Scorpiris* Spach		*I*. *persica* L.	Quaa	April-May	700 m

*Latitude and longitude of sites were not indicated in this table for protection purpose.

**flowering period is generally extended over 3 to 4 weeks. Its starting date may change from year to year according the meteorological conditions.

Rhizomes were then potted in perlite.

We followed the nomenclature of Mouterde published in 1983 [41], although its correspondence with recent nomenclature [42] is also provided in S1 Table. No specific permissions were required for populations located on public lands. Permissions are obtained from religious communities and private land owners for the other populations. Irises are not yet protected in Lebanon. Herbarium specimens are deposited in the National Herbarium at the Lebanese University of Beirut.

### Chromosome preparation and construction of karyotypes

Root tips obtained from rhizomes were pre-treated 24h in 0.05% Colchicine at 4°C or 5h at room temperature. The fixation was performed in Carnoy’s solution (3:1 absolute ethanol: glacial acetic acid) at 4°C for at least 24–48h.

For morphometrical karyotype analysis, the meristems were hydrolyzed in 1 N HCl at 60°C for 12 min, stained in and squashed in 2% (w/v) aceto-orcein. Slides were freezed at -80°C during 24 h, then cover slips were removed, preparations were dried at least 24 h and then mounted in Euparal. Chromosome counts were made on well-spread metaphase plates. The karyotype was determined by examining five metaphase plates per species. Determination of centromere position and chromosome type were made according to Levan *& al*. (1964) [43]. The following characters were measured: long arm (**l**); **s**hort arm (**s**); total chromosome length **(TL)**;; arms ratio (**r** = ratio long/short arms); centromeric index (**Ci%** = 100 x s/TL);; chromosome type (**Ct**; according to Levan *& al*., 1964) [43]; mean centromeric asymmetry (**M**_**CA**_ = Ax100 according to Peruzzi & Eroğlu, 2013) [44]; A = Mean(long arm-short arm)/(long arm+short arm) according to Watanabe *& al*. (1999) [45]; coefficient of variation of chromosome length **CV**_CL_ = *A2* x 100 (Paszko, 2006) [46] where *A2* = standard deviation of chromosome length/mean chromosome length (Romero Zarco, 1986) [47];

### Chromosome preparation for fluorochrome banding and FISH

Chromosome plates for fluorochrome banding and the FISH experiment were prepared using the air-drying technique of [48], with slight modifications. Root tips were washed in a citrate buffer (pH 4.6) for 10 min and then transferred into the enzyme mixture [4% R-10 cellulase (Yakult Honsha Co. Tokyo, Japan), 1% pectolyase Y-23 (Seishin Co. Tokyo, Japan), 4% hemicellulase (Sigma)] in a moist chamber at 37°C for 15 min. The digested meristems were gently squashed in a drop of acetic-acid 45%. Cover slips were removed after freezing over night at -80°C.

For detection of GC-rich DNA regions, chromomycin A_3_ banding (CMA, Sigma) was performed following the technique of [49] with minor modifications [50]: slides were stained with 0.2 μg/ml of CMA solution for 1 h in the dark and mounted in Citifluor AF1 anti-fade agent (Agar Ltd). Some slides stained with chromomycin were destained in fixative (3:1 absolute ethanol:glacial acetic acid), dehydrated in a graded ethanol series (70%, 90%, 100%), air-dried for at least 12 h at room temperature, and then used for the FISH experiment.

A double FISH experiment was carried out with two DNA probes. The 35S rDNA probe was a 4 kb clone from the *Eco*RI fragment, including 18S-5.8S-26S rDNA sequences from *Arabidopsis thaliana* labeled with direct Cy3 fluorochrome (Amersham, Courtaboeuf, France) by nick translation, according to the manufacturer’s protocol. The 5S rDNA probe was the pTa794 clone [51] containing a complete 410 bp *Bam*HI fragment of wheat, including the gene (120 bp) and the spacer (290 bp). The probe was labeled with digoxigenin-11-dUTP (Roche Diagnostics, Meylan, France) after polymerase chain reaction (PCR) using universal M13 primers, and antibody detection was made with anti-digoxigenin-fluorescein (Roche Diagnostics GmbH). The probe mixture consisted of 1–2 ng/μl of each probe, 50% (v/v) formamide, 10% (w/v) dextran-sulfate, 0.1% (w/v) sodium dodecyl sulfate, 250 μg/ml salmon sperm, 20× sodium saline citrate (SSC), completed with ultrapure water. *In situ* hybridization was carried out following the method of [52]. Slides were counterstained and mounted in Vectashield medium containing DAPI (4′, 6-diamidino-2-phenylindole, Vector Laboratories). For rDNA site distribution analyzes, a minimum of 10 well-spread metaphase plates were analyzed for each *Iris* species.

### Microscopy and chromosome analysis

Chromosome observations were performed using an Epifluorescence Zeiss Axiophot microscope with different combinations of excitation and emission filter sets (01, 07, 15 and triple filter set 25). The signals were analyzed using the highly sensitive CCD camera (RETIGA 2000R; Princeton Instruments, Every, France) and an image analyzer (Metavue, Every, France).

## Results

### Chromosome number and karyotype analysis

A diploid chromosome number of 2n = 2x = 20 with basic chromosome number x = 10 was observed in the seven studied *Oncocyclus* taxa. Their detailed karyotypes were established for the first time in this study. Morphometric data for each karyotype are presented in S2 Table. The karyotypes of the *Oncocyclus* taxa were quite similar in size and symmetry (S1 Fig). However, small differences are perceptible at closer look. *I*. *lortetti* for instance is the only taxa among the *Oncocyclus* analyzed to have (2t+18st). Chromosome lengths varied from 1.8 to 6.7 μm.

The remaining non *Oncocyclus* taxa presented cytotypes with different chromosome numbers and ploidy levels: diploid 2n = 2x = 24 for *I*. *persica*, tetraploid 2n = 4x = 40 for *I*. *unguicularis* var. *cretensis* and 2n = 4x = 48 for *I*. *mesopotamica* with basic chromosome numbers of x = 12 and x = 10 respectively. Morphometric data are represented in Table 2.

**Table 2:** Main data on karyotype features of investigated *Iris* taxa.

Taxon	2n (x)	Karyotype formula (2n)	M_CA_	CV_CL_
*I*. *antilibanotica*	20 (2x)	4t+2st-t+14st	71	33
*I*. *bismarckiana*	20 (2x)	10t+10st	73.2	28.1
*I*. *cedreti*	20 (2x)	10t+10st	73.6	31.6
*I*. *lortetii*	20 (2x)	18st+2t	68.4	29.6
*I*. *sofarana* subsp. *sofarana* (Falougha)	20 (2x)	6t+14st	70.6	31.4
*I*. *sofarana* subsp. *sofarana* (Hazzerta)	20 (2x)	6t+14st	71.6	31.7
*I*. *sofarana* subsp. *Kasruwana*	20 (2x)	4t+16st	68.6	29.3
*I*. *westii*	20 (2x)	6t+14st	72.7	30.1
*I*. *mesopotamica*	48 (4x)	24st + 12m+10sm+2sm-st	39.9	20.9
*I*. *unguicularis* var. *cretensis*	40 (4x)	4st+12m+24sm	27.13	24.42
*I*. *persica*	24 (2x)	2st+ 4M-m+12m+6sm	40.4	31.7

M_CA –_ mean centromeric asymmetry [53]_;_ CV_CL –_ coefficient of variation of chromosome length [54].

Total chromosome length of haploid complement varied from 30.5 to 44.5 μm in *Oncocyclus* irises while karyotype symmetry index varied between 84.2% and 86.8%. Total chromosome length and the karyotype symmetry index were respectively 81.2 μm and 60.4% for *I*. *persica*, 74.89 μm and 63.57% for *I*. *unguicularis* var. *cretensis* and 86.7 μm and 43% for *I*. *mesopotamica*.

The mean centromeric asymmetry (M_CA_) of *Oncocyclus* taxa varied from 68.4 to 73.6%. For *I*. *persica*, *I*. *unguicularis* var. *cretensis* and *I*. *mesopotamica*, M_CA_ were 40.4%, 27.13% and 39.9% respectively. The CV_CL_ of the ten taxa, ranged from 20.9% to 33% (Table 2).

### Heterochromatin and ribosomal genes mapping

**Fig 2:**
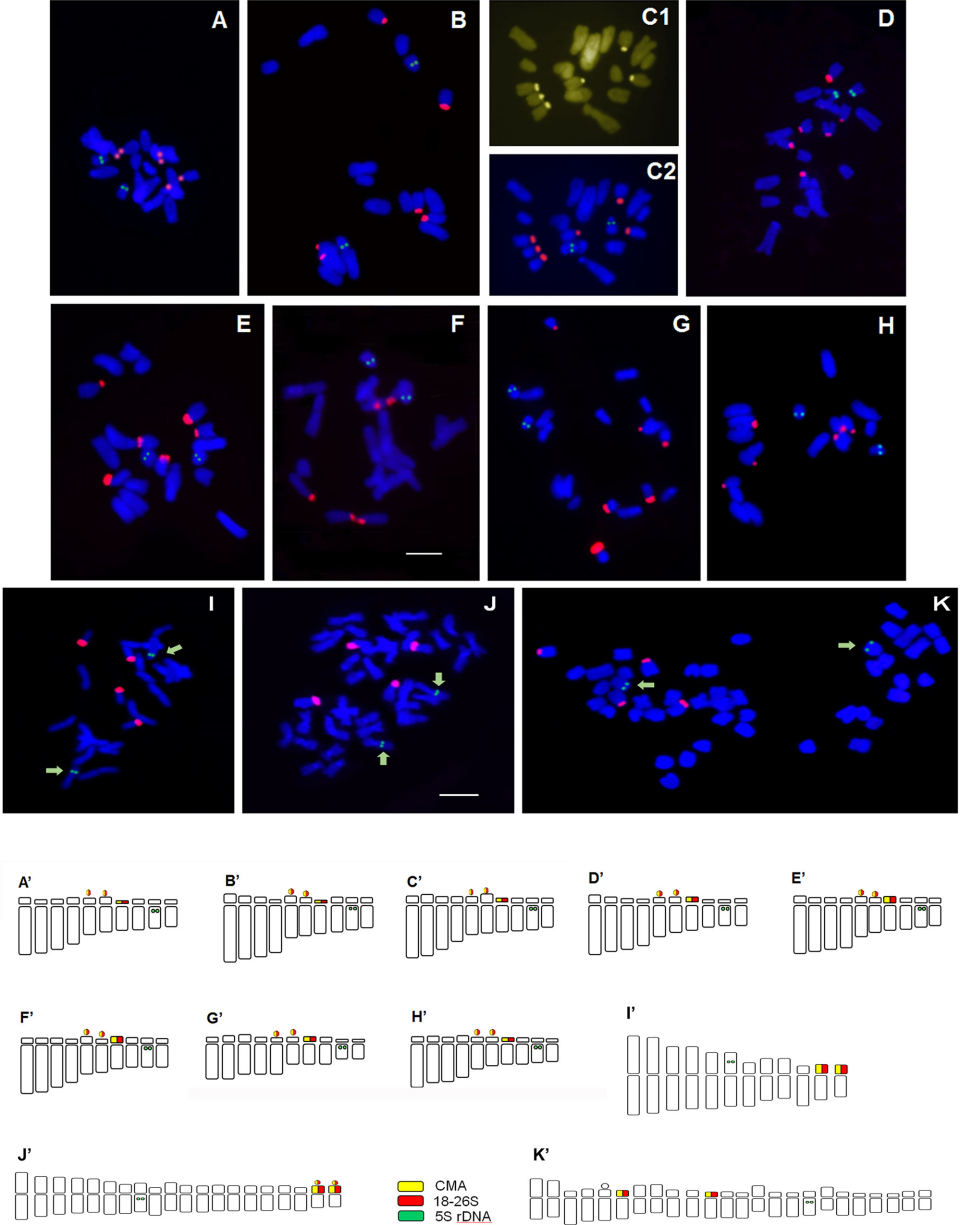
**Metaphase chromosome plates and Idiograms of *Iris* taxa A**–**K**: Metaphase chromosome plates of *Iris* taxa after double target FISH with 5S (green signals) and 18-26S rDNA (red signals) probes. C1 represents CMA staining (yellow signals). **A’**–**K’**: Idiograms with location of 5S (green) and 18-26S rDNA (red) rRNA genes. **A’**: *I*. *sofarana* subsp. *sofarana* (Falougha), **B’**: *I*. *sofarana* subsp. *sofarana* (Hazzerta) **C’**: *I*. *sofarana* subsp. *kasruwana*, **D’**: *I*. *cedreti*, **E’**: *I*. *westii*, **F’**: *I*. *bismarckiana*, **G’**: *I*. *lortetii*, **H’**: *I*. *antilibanotica*, **I’**: *I*. *persica*, **J’**: *I*. *unguicularis* var. *cretensis*, **K’**: *I*. *mesopotamica*. Scale bar 10 *μ*m.

The physical mapping of two rRNA gene families 5S and 35S, and the distributional pattern of GC-rich DNA regions (heterochromatin) in the chromosomes of ten Lebanese taxa have been performed for the first time in this study. The results are presented in Table 3 and Fig 2.

**Table 3:** Synthesis of results concerning molecular cytogenetic approach of investigated *Iris* taxa.

Taxon	2n	Nb and position of CMA^+^ bands	Nb of 35S signals	Nb of 5S signals
*I*. *antilibanotica*	20	6 (5, 6, 7)*	6 (5, 6, 7)*	2 (9)
*I*. *bismarckiana*	20	6 (5, 6, 7)*	6 (5, 6, 7)*	2 (9)
*I*. *cedreti*	20	6 (5, 6, 7)*	6 (5, 6, 7)*	2 (9)
*I*. *lortetii*	20	6 (5, 6, 7)*	6 (5, 6, 7)*	2 (9)
*I*. *sofarana* subsp. *sofarana*	20	6 (5, 6, 7)*	6 (5, 6, 7)*	2 (9)
*I*. *sofarana* subsp. *kasruwana*	20	6 (5, 6, 7)*	6 (5, 6, 7)*	2 (9)
*I*. *westii*	20	6 (5, 6, 7)*	6 (5, 6, 7)*	2 (9)
*I*. *mesopotamica*	48	4 (6, 11)*	4 (10, 11)*	2 (17)
*I*. *unguicularis* var. *cretensis*	40	4 (19, 20)*	4 (19, 20)*	2 (8)
*I*. *persica*	24	4 (11, 12)*	4 (11, 12)*	2 (6)

* Chromosome pair number

Fluorochrome banding revealed GC-rich heterochromatin regions (CMA^+^ bands) which were always associated with 35S rDNA loci. Consequently, the number and position of GC-rich DNA regions correspond to those of 35S loci (Table 3). G-C rich heterochromatin and rDNA organization was conserved and almost identical for all *Oncocyclus* taxa (Fig 2A–2H). The 35S rRNA genes (red signals) were situated in the secondary constrictions (SC) and satellites, which correspond to Nuclear Organized Regions (NORs) of chromosome pair 5 and 6 and on the short arm of chromosome pair 7. Four nucleolar silver-stained granules of active nucleolus organizer regions in interphase cells of *I*. *sofarana* and on *I*. *mesopotamica* were obtained confirming that all the 35S rRNA genes detected by FISH were active in *I*. *mesopotamica* while just 4 out of 6 were active in *I*. *sofarana* (Data not shown). This experiment should be conducted on the other species to confirm this tendency.

In *I*. *persica* ribosomal genes 35S were situated on the short arms of chromosome pairs 11 and 12 (Fig 2I and 2I’), in *I*. *unguicularis* var. *cretensis* on the pairs 19 and 20 (Fig 2J and 2J’), and in *I*. *mesopotamica* on the pairs 6 and 11 (Fig 2K and 2K’). The only 5S locus (green signals) was located intercalary on paracentromeric position of telomeric chromosome pair 9 in all *Oncocyclus* taxa (Fig 2A–2H), on chromosome pair 6 in *I*. *persica*, on pair 8 in *I*. *unguicularis* var. *cretensis* and on pair 17 in *I*. *mesopotamica* (Fig 2I–2K).

## Discussion

### Chromosome number and karyotype features

The karyotypes of investigated taxa were established for the first time using conventional measurements of chromosomes on several metaphase plates per species or population.

Our results show that the karyotype of *Oncocyclus* species remained mainly unchanged. The species showed an extremely similar, bimodal, asymmetric chromosome complement (2n = 20) with four pairs (or five in *I*. *lortetii*) of long chromosomes and six pairs of small ones (Fig 2). Among the long chromosomes, two pairs with a “conspicuous knoblike” short arm and two pairs with “minute”, almost indistinct short arms were observed. The small chromosomes could be differentiated into two satellite pairs, two pairs with knoblike short arms, and two other pairs with minute short arms. All the species from *Oncocyclus* section studied here were found to be diploid with basic chromosome number x = 10 and to present a very close karyotype profile. This is in accordance with results reported by [15,55] for a dozen of *Oncocyclus* taxa analyzed. The constant chromosome number and similar karyotype feature should be considered as a characteristic of the entire section and a diagnostic trait separating the *Oncocylus* from other *Iris* sections.

In addition to the basic chromosome number of x = 10 found in the *Oncocyclus* and *Limniris* sections of the subgenus *Iris*, x = 12 was found in *I*. *mesopotamica* (*Iris* section) and *I*. *persica* (subgenus *Scorpiris*). Basic chromosome numbers of x = 8, 10, 11 and 12 were reported in subgenus *Iris* and x = 10, 11, 12 and 13 in subgenus *Scorpiris* [56]. When comparing basic chromosome number with phylogenetic inferences of *Iris* genus [57], we did not find an explicit trend or correlation between basic chromosomes number and evolutionary history. In each subgenus, different basic chromosome numbers exists. Therefore, we cannot assume neither the smallest nor the biggest basic chromosome number is attributed to the ancestral number.

It is noteworthy that *I*. *persica* taxonomic status in Lebanon is still debated. In fact, Hall & Seisums [58] considered some *Iris persica* populations of Lebanon and Syria as a new species called *Iris wallisiae*. They contradicted Mouterde (1966) who included *I*. *persica* in his treatment of the flora of Syria and Lebanon and declared that there is no evidence of *I*. *persica* ever having been found in Lebanon. Furthermore, according to Hall & Seisums (2014) [58] all reports of *I*. *persica* by Tohmé & Tohmé [59,60,61] for Bekaa valley refer to *I*. *wallisiae*. A chromosome count of 2n = 22 was found by Hall and Seisums (2014) in individuals from three Syrian populations of *I*. *wallisiae* whereas it was 2n = 20 in six Syrian populations of *I*. *persica*. Unexpectedly, our results differ from these chromosome counts, since we found 2n = 24 in the presumed *I*. *persica* population analyzed. Further studies carried out on a larger number of populations in this geographical area need to be conducted to better understand the taxonomy and phytogeography of these taxa.

For *I*. *mesopotamica* we found 2n = 4x = 48 which is in agreement with earlier reports by [62,63]. *Iris mesopotamica* is considered a synonym of *I*. *germanica* [42]. *Iris germanica* was found by Siljak-Yakovlev [64] to be also tetraploid. Both are considered as hybrids and found only in cultivated areas or in the wild having escaped from cultivation. *Iris mesopotamica* is propagated vegetatively and distributed locally, giving the impression of having a stable population with a distinct geographical range [5]. Frequently grown in cemeteries and gardens, it is supposed to come from the Asian Mediterranean coasts (Mouterde, 1983).

For *I*. *unguicularis* var. *cretensis*, we confirm the previous report of 2n = 4x = 40 [65]. This variety is also found in Greece, Crete and Minor Asia. Typical forms of *I*. *unguicularis* are widespread in the Eastern Mediterranean region and North Africa where they display a great variability.

Polyploidy was clearly important in the early diversification of the Iridaceae [66]. In *Iris*, where changes in basic chromosome numbers are common, the ancestral base number, however, remains unclear [66]. The phylogenetic reconstructions do not provide an adequate resolution to interpret neither an increasing nor a decreasing (descending) dysploidy. Although, few studies evaluate dysploidy variation of chromosome number in a phylogenetic framework, a decrease in chromosome numbers comes out not to be unusual [67,68,69,70]. Decreasing dysploidy has been already proposed for several genera of the Iridaceae [66].

Based on the study of the karyotypes feature, we can conclude that *Oncocyclus* is more recent than *Iris*, *Limniris* and *Scorpiris* sections. The constant chromosome number (2n = 20) and extreme similarity of karyotypes among the species of *Oncocyclus* section can be related to its small geographical distribution and shows that *Oncocyclus* is a recent–still evolving–section.

### Distribution pattern of GC-rich DNA regions and rRNA genes

The comparative chromosome mapping of rRNA genes and GC-rich DNA regions have been established here for the first time for all ten investigated taxa. The rDNA is known to participate actively in genome rearrangements [71,72], thus analyzing and comparing 35S and 5S rDNA profiles across related species potentially allow to test hypotheses on their relationships.

Although this kind of studies remains very scarce for the *Iris* genus, the chromosomal locations of 5S and 35S rDNA loci have been previously determined for seven species of subgenus *Xiphium* [40], with a possible trend of increased 35S rDNA loci number during the diversification of the group.

In our study, the two rRNA gene families were located on separate chromosome pairs; thus, the 5S genes are of S-type arrangement [73]. Six signals of 35S rDNA and two signals of 5S rDNA are observed in diploid *Oncocyclus* irises while four signals of 35S and two signals of 5S were detected in tetraploid *I*. *unguicularis* var. *cretenis* and *I*. *mesopotamica*. Polyploidy is frequently associated with epigenetic silencing of rDNA loci [74,75]. Those units and loci that are inactive could be most vulnerable to deletion since their loss would have no selective consequence [76]. In *Iris germanica* which is currently considered as synonym of *I*. *mesopotamica*, Siljak-Yakovlev [64] found eight loci of 35S and two loci of 5S. This different 35S rDNA signals number between these two species questions their real synonymy and call for further studies. Both are tetraploid but their presumed hybrid origin should be confirmed.

Our results prove once again what was reported in previous studies: a loss in 35S and perhaps 5S rDNA loci can be considered as an early indication of genome diploidization in polyploid. In allopolyploids, various types of genetic alterations are observed as reduction in copy number, locus loss, intra- and intergenomic recombination [77,78,79,80]. It was the case for *I*. *versicolor*, an allopolyploid hybrid for which 18–26S rDNA is likely to have undergone a locus loss rather than locus co-evolution [76]. If this should be applied to the polyploidy of *I*. *germanica* and *I*. *mesopotamica*, the first one could be an autopolyploid while the other one could be an interspecific hybrid. Of course, the polyploidy origin should be addressed more carefully by including in the study the putative parents. At this point we can only confirm that ribosomal RNA genes mapping enables the distinction between *I*. *germanica* from Balkans and *I*. *mesopotamica*.

Outlandishly, this technique failed to show differences between the seven *Oncocyclus* irises studied. This finding supports their recent common evolutionary history, as previously suggested [18] Although molecular markers found the two *I*. *sofarana* subspecies as polyphyletic [12], interbreeding between the *Oncocyclus* species is reported to be possible, and creates vital progenies, with fertile hybrids [11]. However, several factors limiting gene flow between populations could have conduced to speciation and selection of the locally adapted genotypes: indeed, (i) the different populations of the Lebanese taxa analyzed in this study are highly fragmented and not close enough for allowing their short flying distance pollinators to maintain gene flow; furthermore, (ii) these taxa are found at different altitudes and flowering dates do not overlap thus contributing to their genetic isolation; finally, (iii) *Oncocyclus* irises are myrmecochory plants, and having their seeds dispersed by ants could also contribute to hinder gene flow. For conservation issues, when population reinforcement will be considered as an option, it is advisable to realize controlled cross-fertilization experiments before in order to confirm their biological compatibility. This kind of information is crucial in order to address IUCN red list status of these taxa.

## Concluding Remarks

This is a contribution to the knowledge of this large genus which has been, until now, rarely studied in this area of research. It provides a first karyomorphological and molecular cytogenetic characterization of ten Lebanese *Iris* species. An important inter-sections variation was detected among the studied taxa. Difference in chromosome number, morphometric data of karyotypes, number and position of GC rich DNA regions and 5S and 35S rDNA loci were evidenced among three species belonging to sections *Iris*, *Limniris* and *Scorpiris*. However the absence of variability and the strong similarity in the karyotype features observed among *Oncocyclus* taxa suggest that this is probably a very young group whose speciation is still in progress as reported in a recent phylogeny including some *Oncocyclus* representative [12]. Additional molecular and cytological studies, including a wider sampling of Oncocyclus irises from the eastern Mediterranean, are currently underway. These studies should clearly describe the diversification and speciation of this section.

## Supporting Information

S1 Fig Metaphase plates and karyotypes of selected Iris taxa stained with acetoorcein (TIF)

S1 Table Accepted names of studied irises and their synonyms according to The Plant List, 2013 (DOCX)

S2 Table Morphometric data concerning the karyotype of *Iris* taxa (DOCX)
