# Supplementary figures and images for: Unlocking the Karyological and Cytogenetic Diversity of Iris from Lebanon: Oncocyclus Section Shows a Distinctive Profile and Relative Stasis during Its Continental Radiation
Source: PLoS One. 2016 Aug 15;11(8):e0160816. doi: 10.1371/journal.pone.0160816 (PMC4985135; doi:10.1371/journal.pone.0160816)

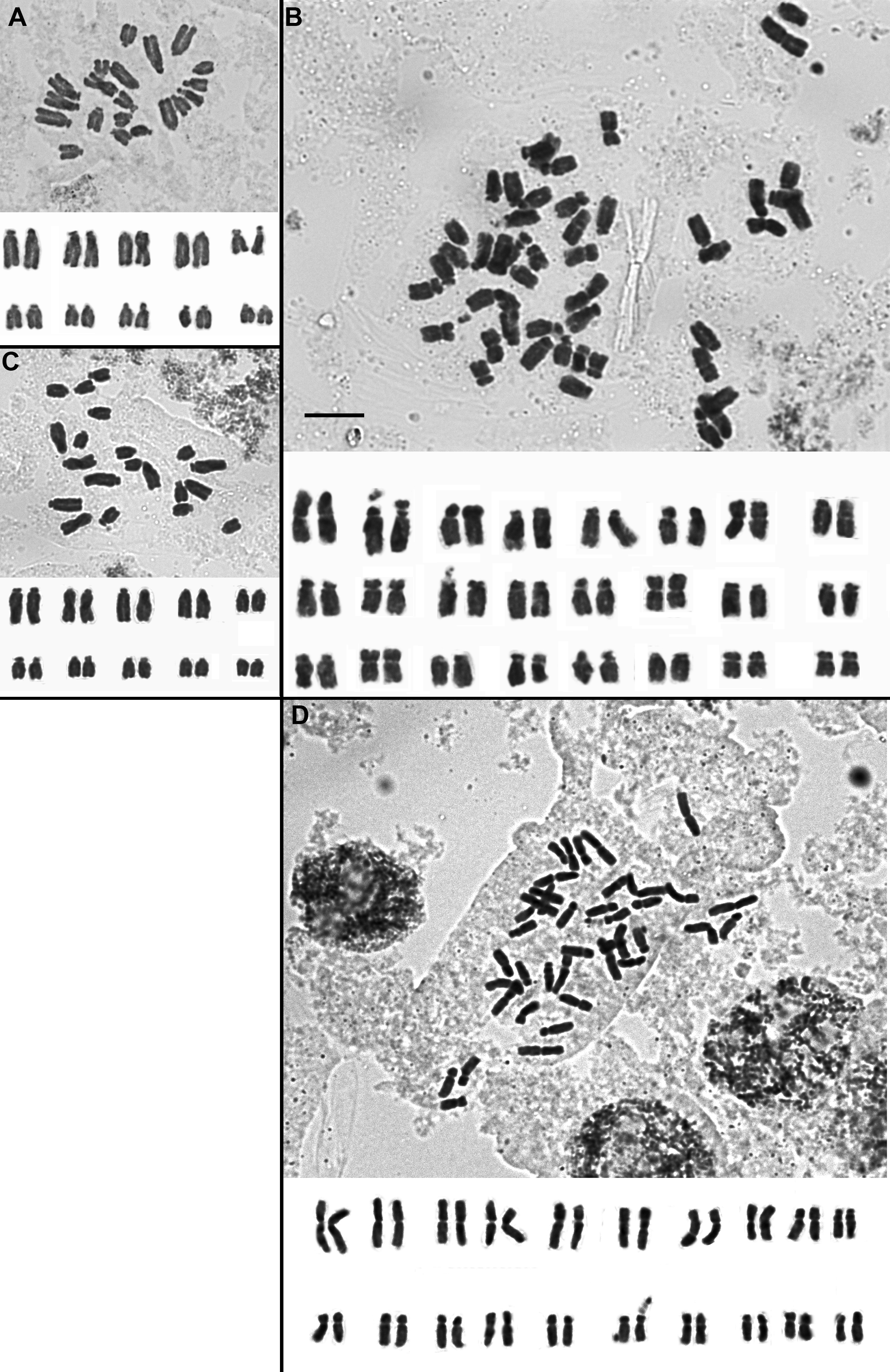

Supplement: S1 Fig — (TIF) [file pone.0160816.s001.tif]
