# Supplementary material for: Unlocking the Karyological and Cytogenetic Diversity of Iris from Lebanon: Oncocyclus Section Shows a Distinctive Profile and Relative Stasis during Its Continental Radiation
Source: PLoS One. 2016 Aug 15;11(8):e0160816. doi: 10.1371/journal.pone.0160816 (PMC4985135; doi:10.1371/journal.pone.0160816)
Supplement: S1 Table — (DOCX) [file pone.0160816.s002.docx]

S1 Table : Accepted names of studied irises and their synonyms according to The Plant List, 2013 (42).

| **Scientific name given by Mouterde** | **Accepted scientific name** | **Synonyms** | **Infraspecific taxon** | **Synonyms** |
| --- | --- | --- | --- | --- |
| *Iris westii* Dinsm. | *Iris westii* Dinsm. | *Iris sofarana* f. *westii* |  |  |
|  |  | *Iris susiana* f. *westii* |  |  |
| *Iris cedreti* Dinsm. ex Chaudhary | *Iris cedreti* Dinsm. ex Chaudhary |  |  |  |
| *Iris sofarana* Foster | *Iris susiana* L. | *Iris grandiflora* Salisb. |  |  |
| *Iris sofarana* subsp. *kasruwana* |  | *Iris kasruwana* Dinsm. |  |  |
|  |  | *Iris livida* Tratt. |  |  |
|  |  | *Iris punctata* Moench |  |  |
|  |  | *Iris sofarana* Foster |  |  |
|  |  | *Iris sofarana* f. *franjieh* |  |  |
|  |  | *Iris sofarana* f. *kasruwana* |  |  |
|  |  | *Iris sofarana* subsp. *kasruwana* |  |  |
|  |  | *Iris sofarana* var. *magnifica* |  |  |
|  |  | *Oncocyclus susianus* (L.) K.Koch |  |  |
| *Iris lortetii* Barbey ex Boiss. | *Iris lortetii* Barbey ex Boiss. |  | *Iris lortetii* var. *lortetii* | *Iris lortetii* var. *samariae* |
|  |  |  |  | *Iris samariae* Dinsm. |
| *Iris antilibanotica* Dinsm. | *Iris antilibanotica* Dinsm. |  |  |  |
| *Iris bismarckiana* Damman & Sprenger | *Iris bismarckiana* Damman & Sprenger | *Iris nazerena* (Foster) Dinsm. |  |  |
|  |  | *Iris sari* var. *nazerena* |  |  |
|  |  | *Iris sari-nazarens* Foster |  |  |
| *Iris persica* L. | *Iris persica* L. | *Coresantha persica* (L.) Alef. |  |  |
|  |  | *Costia persica* (L.) Willk. |  |  |
|  |  | *Iris bolleana* Siehe |  |  |
|  |  | *Iris haussknechtii* Siehe |  |  |
|  |  | *Iris issica* Siehe |  |  |
|  |  | *Iris persica* var. *bolleana* |  |  |
|  |  | *Iris persica* var. *isaacsonii* |  |  |
|  |  | *Iris persica* var. *issica* |  |  |
|  |  | *Iris persica* var. *purpurea* |  |  |
|  |  | *Iris persica* f. *sieheana* |  |  |
|  |  | *Iris persica* var. *sieheana* |  |  |
|  |  | *Iris praecox* Salisb. |  |  |
|  |  | *Iris purpurea* Siehe |  |  |
|  |  | *Iris sieheana* Lynch |  |  |
|  |  | *Iris squamata* Gaterau |  |  |
|  |  | *Juno persica* (L.) Tratt. |  |  |
|  |  | *Thelysia persica* (L.) Parl. |  |  |
|  |  | *Xiphion persicum* (L.) Mill. |  |  |
| *Iris unguicularis* subsp. *cretensis* | *Iris unguicularis* subsp. *cretensis* | *Iris cretensis* Janka |  |  |
|  |  | *Iris cretica* Herb. ex Baker |  |  |
|  |  | *Iris humilis* Sieber ex Baker |  |  |
|  |  | *Iris humilis* subsp. *cretensis* |  |  |
|  |  | *Siphonostylis cretensis* (Janka) Wern.Schulze |  |  |

*** The Plant List (2013). Version 1.1. Published on the Internet; http://www.theplantlist.org/ (accessed November 2015).**
