# Supplementary material for: Unlocking the Karyological and Cytogenetic Diversity of Iris from Lebanon: Oncocyclus Section Shows a Distinctive Profile and Relative Stasis during Its Continental Radiation
Source: PLoS One. 2016 Aug 15;11(8):e0160816. doi: 10.1371/journal.pone.0160816 (PMC4985135; doi:10.1371/journal.pone.0160816)
Supplement: S2 Table — (DOCX) [file pone.0160816.s003.docx]

**S2 Table**

Morphometric data concerning the karyotype of *Iris* taxa **l -**long arm; **s** -short arm; **TL** -Total chromosome length; **r** -ratio long/short arms; **Ci%** -Centromeric index =100 x s/TL**;**; **Ct** -Chromosome type (according to Levan *& al*., 1964); **M_CA_** -Mean Centromeric Asymmetry= Ax100  [A = Mean (long arm-short arm)/(long arm+short arm)]; **CV_CL_** -Coefficient of Variation of Chromosome Length = A2 x 100  [A2= standard deviation of chromosome length/mean chromosome length (Romero Zarco 1986)]; **m** -metacentric; **sm** –submetacentric; **sat** -satellite **SD** -Standard Deviation. All the values are in μm.

**12k**

| **Chr.p** | **s** | **l** | **TL=l+s** | **r=l/s** | **Ci%=100s/TL** | **Ct** | **M_CA_%** | **CV_CL_%** |
| --- | --- | --- | --- | --- | --- | --- | --- | --- |
| **1** | **0.6** | **5** | **5.6** | **8.3** | **10.7** | **t** | **70.6** | **31.4** |
| **2** | **0.5** | **4.8** | **5.3** | **9.6** | **9.4** | **t** |  |  |
| **3** | **0.5** | **4.3** | **4.8** | **8.6** | **10.4** | **t** |  |  |
| **4** | **0.5** | **3.8** | **4.3** | **7.6** | **11.6** | **st** |  |  |
| **5** | **0.6** | **2.8** | **3.4** | **4.7** | **17.6** | **st-sat** |  |  |
| **6** | **0.7** | **2.5** | **3.2** | **3.6** | **21.9** | **st-sat** |  |  |
| **7** | **0.6** | **2.3** | **2.9** | **3.8** | **20.7** | **st** |  |  |
| **8** | **0.5** | **2.3** | **2.8** | **4.6** | **17.9** | **st** |  |  |
| **9** | **0.5** | **2.1** | **2.6** | **4.2** | **19.2** | **st** |  |  |
| **10** | **0.5** | **2** | **2.5** | **4.0** | **20.0** | **st** |  |  |
| **Total** | **5.5** | **32** | **37.4** |  |  |  |  |  |
| **Average** | **0.5** | **3.2** | **3.7** |  |  |  |  |  |
| **SD** | **0.1** | **1.2** | **1.2** |  |  |  |  |  |

1. ***sofarana* subsp. *sofarana* (Falougha)**

| **Chr.p** | **s** | **l** | **TL=l+s** | **r=l/s** | **Ci%=100s/TL** | **Ct** | **MCA%** | **CV_CL_%** |
| --- | --- | --- | --- | --- | --- | --- | --- | --- |
| **1** | **0.9** | **5.8** | **6.7** | **6.3** | **13.7** | **st** | **71.6** | **31.7** |
| **2** | **0.7** | **5.5** | **6.2** | **7.9** | **11.3** | **t** |  |  |
| **3** | **0.6** | **5.3** | **5.8** | **8.9** | **10.1** | **t** |  |  |
| **4** | **0.4** | **4.9** | **5.2** | **13.6** | **6.9** | **t** |  |  |
| **5** | **0.7** | **3.3** | **4.0** | **4.8** | **17.3** | **st-sat** |  |  |
| **6** | **0.6** | **3.1** | **3.7** | **5.5** | **15.4** | **st-sat** |  |  |
| **7** | **0.8** | **2.9** | **3.7** | **3.6** | **21.7** | **st** |  |  |
| **8** | **0.6** | **2.7** | **3.3** | **4.4** | **18.6** | **st** |  |  |
| **9** | **0.6** | **2.5** | **3.1** | **4.5** | **18.3** | **st** |  |  |
| **10** | **0.5** | **2.3** | **2.8** | **4.3** | **18.9** | **st** |  |  |
| **Total** | **6.3** | **38.2** | **44.5** | **63.7** |  |  |  |  |
| **Average** | **0.6** | **3.8** | **4.5** |  |  |  |  |  |
| **SD** |  |  | **1.4** |  |  |  |  |  |

***I. sofarana* subsp. *sofarana* (Hazzerta)**

| **Chr.p** | **s** | **l** | **TL=l+s** | **r=l/s** | **Ci%=100s/TL** | **Ct** | **MCA%** | **CV_CL_%** |
| --- | --- | --- | --- | --- | --- | --- | --- | --- |
| **1** | **0.7** | **5.4** | **6.1** | **7.6** | **11.6** | **t** | **68.6** | **29.3** |
| **2** | **0.6** | **5.1** | **5.7** | **8.9** | **10.1** | **t** |  |  |
| **3** | **0.7** | **4.5** | **5.2** | **6.1** | **14.1** | **st** |  |  |
| **4** | **0.6** | **3.7** | **4.3** | **5.7** | **14.9** | **st** |  |  |
| **5** | **0.6** | **3.0** | **3.6** | **4.7** | **17.6** | **st-sat** |  |  |
| **6** | **0.9** | **2.8** | **3.7** | **3.1** | **24.1** | **st-sat** |  |  |
| **7** | **0.6** | **2.7** | **3.3** | **4.4** | **18.4** | **st** |  |  |
| **8** | **0.6** | **2.6** | **3.1** | **4.4** | **18.4** | **st** |  |  |
| **9** | **0.5** | **2.5** | **3.0** | **4.8** | **17.1** | **st** |  |  |
| **10** | **0.5** | **2.2** | **2.7** | **4.3** | **18.8** | **st** |  |  |
| **Total** | **6.4** | **34.4** | **40.9** |  |  |  |  |  |
| **Average** | **0.6** | **3.4** | **4.1** |  |  |  |  |  |
| **SD** |  |  | **1.2** |  |  |  |  |  |

***I. sofarana* subsp*. kasruwana***

| **Chr. p** | **s** | **l** | **TL=l+s** | **r=l/s** | **Ci%=100s/TL** | **Ct** | **M_CA_%** | **CV_CL_%** |
| --- | --- | --- | --- | --- | --- | --- | --- | --- |
| **1** | **0.8** | **5.1** | **5.9** | **6.4** | **13.5** | **st** | **73.6** | **31.6** |
| **2** | **0.6** | **4.7** | **5.3** | **7.6** | **11.6** | **t** |  |  |
| **3** | **0.4** | **4.5** | **4.9** | **10.9** | **8.4** | **t** |  |  |
| **4** | **0.4** | **4.1** | **4.5** | **11.5** | **8.0** | **t** |  |  |
| **5** | **0.5** | **3.2** | **3.7** | **6.2** | **14.0** | **st-sat** |  |  |
| **6** | **0.6** | **2.7** | **3.3** | **4.7** | **17.7** | **st-sat** |  |  |
| **7** | **0.6** | **2.6** | **3.1** | **4.5** | **18.0** | **st** |  |  |
| **8** | **0.5** | **2.3** | **2.8** | **4.3** | **18.9** | **st** |  |  |
| **9** | **0.3** | **2.2** | **2.5** | **7.2** | **12.2** | **t** |  |  |
| **10** | **0.4** | **2.1** | **2.4** | **5.3** | **15.8** | **t** |  |  |
| **Total** | **5.1** | **33.4** | **38.5** |  |  |  |  |  |
| **Average** | **0.5** | **3.3** | **3.8** |  |  |  |  |  |
| **SD** |  |  | **1.2** |  |  |  |  |  |

***I. cedreti***

| **Chr.p** | **s** | **l** | **TL=l+s** | **r=l/s** | **Ci%=100s/TL** | **Ct** | **M_CA_%** | **CV_CL_%** |
| --- | --- | --- | --- | --- | --- | --- | --- | --- |
| **1** | **0.7** | **5.0** | **5.7** | **6.7** | **12.9** | **st** | **72.7** | **30.1** |
| **2** | **0.5** | **4.7** | **5.2** | **9.8** | **9.3** | **t** |  |  |
| **3** | **0.5** | **4.5** | **5.0** | **8.8** | **10.2** | **t** |  |  |
| **4** | **0.5** | **4.4** | **4.8** | **9.1** | **9.9** | **t** |  |  |
| **5** | **0.7** | **3.2** | **3.9** | **4.8** | **17.4** | **st-sat** |  |  |
| **6** | **0.5** | **2.8** | **3.3** | **5.8** | **14.8** | **st-sat** |  |  |
| **7** | **0.7** | **2.6** | **3.3** | **3.6** | **21.6** | **st** |  |  |
| **8** | **0.4** | **2.5** | **2.9** | **5.9** | **14.4** | **st** |  |  |
| **9** | **0.4** | **2.2** | **2.6** | **4.9** | **17.1** | **st** |  |  |
| **10** | **0.4** | **2.1** | **2.5** | **4.9** | **16.9** | **st** |  |  |
| **Total** | **5.4** | **33.8** | **39.2** |  |  |  |  |  |
| **Average** | **0.5** | **3.4** | **3.9** |  |  |  |  |  |
| **SD** |  |  | **1.2** |  |  |  |  |  |

***I. westii***

| **Chr.p** | **s** | **l** | **TL=l+s** | **r=l/s** | **Ci%=100s/TL** | **Ct** | **M_CA_%** | **CV_CL_%** |
| --- | --- | --- | --- | --- | --- | --- | --- | --- |
| **1** | **0.5** | **5.0** | **5.5** | **9.8** | **9.3** | **t** | **73.2** | **28.1** |
| **2** | **0.5** | **4.8** | **5.3** | **9.4** | **9.6** | **t** |  |  |
| **3** | **0.5** | **4.4** | **4.9** | **8.5** | **10.5** | **t** |  |  |
| **4** | **0.4** | **3.8** | **4.2** | **10.0** | **9.1** | **t** |  |  |
| **5** | **0.6** | **2.9** | **3.6** | **4.6** | **17.9** | **st-sat** |  |  |
| **6** | **0.4** | **2.8** | **3.2** | **7.3** | **12.0** | **t-sat** |  |  |
| **7** | **0.6** | **2.4** | **3.1** | **3.8** | **20.8** | **st** |  |  |
| **8** | **0.6** | **2.3** | **2.9** | **4** | **20.0** | **st** |  |  |
| **9** | **0.5** | **2.3** | **2.8** | **4.5** | **18.2** | **st** |  |  |
| **10** | **0.4** | **2.3** | **2.8** | **5.1** | **16.3** | **st** |  |  |
| **Total** | **5.1** | **33.1** | **38.3** |  |  |  |  |  |
| **Average** | **0.5** | **3.3** | **3.8** |  |  |  |  |  |
| **SD** |  |  | **1.1** |  |  |  |  |  |

***I. bismarckiana***

| **Chr.p** | **s** | **l** | **TL=l+s** | **r=l/s** | **Ci=100s/TL** | **Ct** | **M_CA_%** | **CV_CL_%** |
| --- | --- | --- | --- | --- | --- | --- | --- | --- |
| **1** | **0.6** | **3.6** | **4.2** | **6** | **14.3** | **st** | **68.4** | **29.6** |
| **2** | **0.5** | **3.6** | **4.1** | **5.6** | **12.2** | **st** |  |  |
| **3** | **0.8** | **3.1** | **3.8** | **4** | **20.0** | **st** |  |  |
| **4** | **0.5** | **3.1** | **3.6** | **6** | **14.3** | **st** |  |  |
| **5** | **0.4** | **3.1** | **3.5** | **8** | **11.1** | **t-sat** |  |  |
| **6** | **0.5** | **2.1** | **2.6** | **4** | **20.0** | **st-sat** |  |  |
| **7** | **0.5** | **2.1** | **2.6** | **4** | **20.0** | **st** |  |  |
| **8** | **0.5** | **2.1** | **2.6** | **4** | **20.0** | **st** |  |  |
| **9** | **0.3** | **1.5** | **1.8** | **6** | **14.3** | **st** |  |  |
| **10** | **0.3** | **1.5** | **1.8** | **6** | **14.3** | **st** |  |  |
| **Total** | **4.9** | **25.6** | **30.5** |  |  |  |  |  |
| **Average** | **0.3** | **1.5** | **3** |  |  |  |  |  |
| **SD** |  |  | **0.9** |  |  |  |  |  |

***I. lortetii***

| **Chr.p** | **s** | **l** | **TL=l+s** | **r=l/s** | **Ci=100s/TL** | **Ct** | **M_CA_%** | **CV_CL_%** |
| --- | --- | --- | --- | --- | --- | --- | --- | --- |
| **1** | **0.56** | **4.44** | **5** | **8** | **11** | **t** | **71** | **33** |
| **2** | **0.60** | **3.93** | **5** | **7** | **13** | **st-t** |  |  |
| **3** | **0.47** | **3.85** | **4** | **8** | **11** | **t** |  |  |
| **4** | **0.64** | **3.42** | **4** | **5** | **16** | **st** |  |  |
| **5** | **0.51** | **2.56** | **3** | **5** | **17** | **st-sat** |  |  |
| **6** | **0.51** | **2.22** | **3** | **4** | **19** | **st-sat** |  |  |
| **7** | **0.43** | **2.05** | **2** | **5** | **17** | **st** |  |  |
| **8** | **0.38** | **2.05** | **2** | **5** | **16** | **st** |  |  |
| **9** | **0.34** | **1.88** | **2** | **6** | **15** | **st** |  |  |
| **10** | **0.30** | **1.79** | **2** | **6** | **14** | **st** |  |  |
| **Total** | **5** | **28** | **33** |  |  |  |  |  |
| **Average** | **0** | **3** | **3** |  |  |  |  |  |
| **SD** |  |  | **1** |  |  |  |  |  |

***I. antilibanotica***

| **Chr.p** | **s** | **l** | **TL=l+s** | **r=l/s** | **Ci=100s/TL** | **Ct** | **M_CA_%** | **CV_CL_%** |
| --- | --- | --- | --- | --- | --- | --- | --- | --- |
| **1** | **5.1** | **5.3** | **10.5** | **1** | **49** | **m** | **40.4** | **31.7** |
| **2** | **4.9** | **5.1** | **10.1** | **1** | **49** | **m** |  |  |
| **3** | **3.7** | **4.7** | **8.4** | **1.3** | **43.9** | **m** |  |  |
| **4** | **3.7** | **4.5** | **8.2** | **1.2** | **45** | **m** |  |  |
| **5** | **2.9** | **4.5** | **7.4** | **1.6** | **38.9** | **m** |  |  |
| **6** | **2.9** | **4.1** | **7.0** | **1.4** | **41.2** | **m** |  |  |
| **7** | **2.1** | **3.3** | **5.3** | **1.6** | **38.5** | **m** |  |  |
| **8** | **2.1** | **3.1** | **5.1** | **1.5** | **40** | **m** |  |  |
| **9** | **1.4** | **4.1** | **5.5** | **2.9** | **25** | **sm** |  |  |
| **10** | **1** | **4.1** | **5.1** | **4** | **20** | **st** |  |  |
| **11** | **1.2** | **3.3** | **4.5** | **2.7** | **27.3** | **sm** |  |  |
| **12** | **1.2** | **2.9** | **4.1** | **2.3** | **30** | **sm** |  |  |
| **Total** | **32.2** | **49** | **81.2** |  |  |  |  |  |
| **Average** | **2.7** | **4.1** | **6.8** |  |  |  |  |  |
| **SD** |  |  | **2.1** |  |  |  |  |  |

***I.*** ***persica***

| **Chr.p** | **s** | **l** | **TL=l+s** | **r=l/s** | **Ci%=100s/TL** | **Ct** | **M_CA_%** | **CV_CL_%** |
| --- | --- | --- | --- | --- | --- | --- | --- | --- |
| **1** | **2.8** | **3.38** | **6.20** | **1.20** | **45.5** | **m** | **27.13** | **24.42** |
| **2** | **2.3** | **2.95** | **5.26** | **1.28** | **43.9** | **m** |  |  |
| **3** | **2.1** | **2.65** | **4.79** | **1.24** | **44.6** | **m** |  |  |
| **4** | **2.1** | **2.52** | **4.57** | **1.23** | **44.9** | **m** |  |  |
| **5** | **2.0** | **2.39** | **4.36** | **1.22** | **45.1** | **m** |  |  |
| **6** | **1.5** | **2.65** | **4.19** | **1.72** | **36.7** | **sm** |  |  |
| **7** | **1.4** | **2.35** | **3.72** | **1.72** | **36.8** | **sm** |  |  |
| **8** | **1.5** | **2.22** | **3.68** | **1.53** | **39.5** | **m** |  |  |
| **9** | **1.2** | **2.56** | **3.76** | **2.14** | **31.8** | **sm** |  |  |
| **10** | **0.8** | **2.82** | **3.59** | **3.67** | **21.4** | **st** |  |  |
| **11** | **1.0** | **2.35** | **3.38** | **2.29** | **30.4** | **sm** |  |  |
| **12** | **1.0** | **2.35** | **3.38** | **2.29** | **30.4** | **sm** |  |  |
| **13** | **1.1** | **2.22** | **3.33** | **2.00** | **33.3** | **sm** |  |  |
| **14** | **0.9** | **2.14** | **3.08** | **2.27** | **30.6** | **sm** |  |  |
| **15** | **1.0** | **2.18** | **3.21** | **2.13** | **32.0** | **sm** |  |  |
| **16** | **0.7** | **2.18** | **2.88** | **3.09** | **24.4** | **st** |  |  |
| **17** | **1.0** | **2.18** | **3.21** | **2.13** | **32.0** | **sm** |  |  |
| **18** | **1.0** | **2.05** | **3.08** | **2.00** | **33.3** | **sm** |  |  |
| **19** | **1.0** | **1.79** | **2.82** | **1.75** | **36.4** | **sm** |  |  |
| **20** | **0.8** | **1.67** | **2.44** | **2.17** | **31.6** | **sm** |  |  |
| **Total** | **27.29** | **47.61** | **74.89** |  |  |  |  |  |
| **Average** | **1.36** | **2.38** | **3.74** |  |  |  |  |  |
| **SD** |  |  | **0.91** |  |  |  |  |  |

***I. unguicularis* var. *cretensis***

| **Chr.p** | **s** | **l** | **TL=l+s** | **r=l/s** | **Ci%=100s/TL** | **Ct** | **M_CA_%** | **CV_CL_%** |
| --- | --- | --- | --- | --- | --- | --- | --- | --- |
| **1** | **2.7** | **3.0** | **5.6** | **1.1** | **47.3** | **m** | **39.9** | **20.9** |
| **2** | **2.4** | **2.7** | **5.0** | **1.1** | **46.9** | **m** |  |  |
| **3** | **1.6** | **2.2** | **3.8** | **1.3** | **43.2** | **m** |  |  |
| **4** | **1.7** | **2.1** | **3.8** | **1.2** | **45.9** | **m** |  |  |
| **5** | **1.6** | **1.8** | **3.4** | **1.2** | **46.3** | **m** |  |  |
| **6** | **1.4** | **1.6** | **3.1** | **1.1** | **46.7** | **m** |  |  |
| **7** | **0.8** | **3.8** | **4.6** | **4.6** | **17.8** | **st** |  |  |
| **8** | **1.1** | **3.3** | **4.4** | **2.9** | **25.6** | **sm** |  |  |
| **9** | **1.0** | **3.2** | **4.2** | **3.1** | **24.4** | **st** |  |  |
| **10** | **0.9** | **3.0** | **3.8** | **3.4** | **22.7** | **st** |  |  |
| **11** | **0.7** | **2.9** | **3.6** | **4.0** | **20.0** | **st** |  |  |
| **12** | **0.6** | **2.9** | **3.5** | **4.7** | **17.6** | **st** |  |  |
| **13** | **0.8** | **2.8** | **3.6** | **3.4** | **22.9** | **st** |  |  |
| **14** | **1.0** | **2.7** | **3.7** | **2.6** | **27.8** | **sm** |  |  |
| **15** | **0.9** | **2.7** | **3.6** | **2.9** | **25.7** | **sm** |  |  |
| **16** | **0.7** | **2.7** | **3.4** | **3.7** | **21.2** | **st** |  |  |
| **17** | **0.7** | **2.7** | **3.4** | **3.7** | **21.2** | **st** |  |  |
| **18** | **0.7** | **2.5** | **3.2** | **3.4** | **22.6** | **st** |  |  |
| **19** | **0.8** | **2.5** | **3.3** | **3.0** | **25.0** | **sm-st** |  |  |
| **20** | **0.5** | **2.5** | **3.0** | **4.8** | **17.2** | **st** |  |  |
| **21** | **0.9** | **2.1** | **3.0** | **2.2** | **31.0** | **st** |  |  |
| **22** | **0.5** | **2.1** | **2.6** | **4.0** | **20.0** | **st** |  |  |
| **23** | **0.8** | **1.7** | **2.6** | **2.1** | **32.0** | **sm** |  |  |
| **24** | **0.9** | **1.6** | **2.6** | **1.8** | **36.0** | **sm** |  |  |
| **Total** | **26.1** | **60.6** | **86.7** |  |  |  |  |  |
| **Average** | **1.1** | **2.5** | **3.6** |  |  |  |  |  |
| **SD** |  |  | **0.8** |  |  |  |  |  |

***I. mesopotamica***
